# The Effect of Coronavirus Disease 2019 on the Quality of Associated Care in Patients with Gastric Cancer

**DOI:** 10.34172/mejdd.2024.363

**Published:** 2024-01-31

**Authors:** Navid Omidifar, Nasrin Pazoki, Mansoureh Shokripour, Mohammad Reza Fattahi, Ali Reza Safarpour, Ebrahim Fallahzadeh Abarghooee, Nika Nikmanesh, Seyedeh Azra Shamsdin, Hassan Akrami, Seyyed Amirreza Saghi, Yousef Nikmanesh

**Affiliations:** ^1^Biotechnology Research Center and Department of Pathology, Medical School, Shiraz University of Medical Sciences, Shiraz, Iran; ^2^Department of Genetics, Faculty of Biological Sciences, North Tehran Branch, Islamic Azad University, Tehran, Iran; ^3^Department of Pathology, School of Medicine, Shiraz University of Medical Sciences, Shiraz, Iran; ^4^Gastroenterohepatology Research Center, Shiraz University of Medical Sciences, Shiraz, Iran; ^5^Department of Internal Medicine, School of Medicine, Shiraz University of Medical Sciences, Shiraz, Iran; ^6^Cellular and Molecular Biology Research Center, Larestan University of Medical Sciences, Larestan, Iran; ^7^Student Research Committee, Larestan University of Medical Sciences, Larestan, Iran

**Keywords:** COVID-19, Coronavirus, Gastric, Stomach, Cancer, Quality of care

## Abstract

Coronavirus is a new virus that has affected human life on a large scale; it has infected millions of people and killed hundreds of thousands of people. In contrast, among cancers, stomach neoplasia is the most common cancer of the upper gastrointestinal (UGI) tract. COVID-19 disease has disrupted the optimal management of patients with cancer. Metastasis, deterioration of the patient’s nutritional status, UGI bleeding, and increased surgical complications are all consequences of delayed treatment of patients with gastric cancer. However, there is still insufficient evidence on the immunogenicity of the vaccine and the protection provided by coronavirus vaccines in patients with cancer, especially those with immunodeficiency or those who are treated for certain types of cancers. Also, as part of the prevention and control of COVID-19 disease, nutritional support for patients with gastrointestinal cancer is particularly important, and the psychological and physiological limitations caused by the disease duration are hurting the well-being of patients. Therefore, the assessment of the impact of the coronavirus on cancer should be treated as an important issue, and healthcare professionals should be prepared to deal with the long-term effects of the coronavirus disease.

## Introduction

 A novel virus called SARS-CoV-2 that caused an acute respiratory illness broke out in Wuhan, China, in 2019.^1^ This disease spread in the cities of China and other countries. The severity of the disease caused the World Health Organization (WHO) to declare an international emergency related to this disease on January 30, 2020. On February 12, 2020, the disease was named COVID-19.^2,3^ The contagiousness of this disease was very high, so it quickly became an epidemic and spread to all continents except Antarctica.^4^

 The most commonly reported symptoms are fever and fatigue, which then lead to difficulty breathing and, in advanced cases, can be fatal. However, combined or isolated symptoms of abdominal and gastrointestinal disorders such as diarrhea, nausea, vomiting, and abdominal pain have been reported.^5,6^

 Considering the different and unpredictable symptoms of COVID-19 in patients, the development of this disease in people with immune system defects and cancers is very anxiety. Since the COVID-19 disease can also target the digestive system, the occurrence of this disease in people with abdominal cancers such as colon cancer or gastric cancer is very important and should be considered.^7^

 Evidence suggested that about 55% of the most commonly diagnosed cancers worldwide in 2012 were lung, breast, colorectal, prostate, gastric, and liver cancers.^8^ Based on the results of articles published so far, gastric cancer is one of the most common types of cancers, also known as upper gastrointestinal (UGI) cancer.^9^

 As reported by GLOBOCAN in 2020, stomach cancer was the fifth most common type of cancer. The estimate of one million new cases diagnosed in the world indicates a significant increase in the incidence of this cancer in recent years.^10^ Among all common cancers, gastric cancer is the third most common cause of cancer-related deaths worldwide among men and women (about 723 100 deaths). The high prevalence of COVID-19 has disrupted the care of cancer patients. Lack of medical care and regular services such as endoscopy and early cancer detection have been reported as some of the problems caused by the corona infection in the treatment of cancer patients in England.^11,12^

 One of the most important reasons for the disruption in the process of diagnosing and treating patients with gastric cancer was the decrease in the number of hospitalized patients due to the spread of Corona.^13,14^ In other words, delays in treatment and lack of follow-up due to fear of COVID-19 resulted in adverse health effects for patients across medical and surgical paradigms.^15^ Other impacts of COVID-19 on the treatment process of patients with gastric cancer include metastasis, deterioration of the patient’s nutritional status, bleeding from the UGI tract, and amplification of surgical complications.^16^ Considering the importance of diagnosing and quickly following up on gastric cancer, it is necessary to pay attention of appropriate treatment measures during the coronavirus infection period and its limitations.^17^

## A New Approach to Pathogenesis

 Pathophysiological studies of COVID-19 and associated cellular pathways involved in viral invasion, replication, and immunogenesis not only aid in finding a cure for the disease but also play an important role in the investigation and classification of this disease.^2,18^

**Figure 1:**
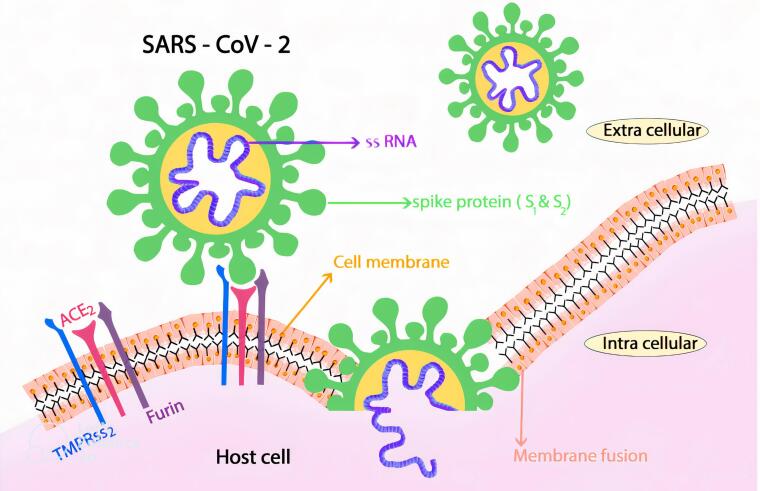
The host cell entry stages of SARS-Cov2, schematic reaction between SARS-Cov2 surface protein (S1 and S2) and angiotensin-converting enzyme 2 (ACE2) receptor at the host cell. It is noteworthy that this connection is efficient when the transmembrane serine protease 2 (TMPRSS2) and furan molecules are also involved in the process

 One of the initial hypotheses for the potential pathogenesis of SARS-CoV-2 infection was that the angiotensin-converting enzyme-2 (ACE-2) receptor provides the entry point for the coronavirus to hook into and infect a wide range of human cells ^19^ (Figure 1).

 ACE-2 is a type 1 membrane protein that is active in cells of the epithelial lineage of various tissues, including the lung, gastrointestinal tract, liver, kidney, and brain. ACE-2, an important part of the renin-angiotensin system, is known not only for its role in regulating blood pressure by hydrolyzing and converting angiotensin II to angiotensin,^1-7^ but also it plays a role in many physiological processes such as inflammation, repair tissue, and neurodegeneration.^20,21^

 ACE-2 has been recognized as an active host receptor for the introduction of the novel SARS-CoV-2 illness into pulmonary alveolus.^22^ Due to the great structural similarity between SARS-CoV-1 and SARS-CoV-2, it may be decided that ACE-2 is a deliberate potential basic introduction route for SARS-CoV-2 infection at the beginning of COVID-19 disrase.^23^ SARS-CoV-2 viruses come to target cell through a surface pierce (S) protein. On the other hand, SARS-CoV-2 is generally cleaved for one host sheet, serine protease 2 (TMPRSS2), and the disinterring and metalloproteinase 17 ADAM17.^24^ The simultaneous action of ACE-2 and TMPRSS2 can create and facilitate the access path of SARS-CoV-2 to the gastrointestinal tract not only in pneumocytes but also in the lymphatic enterocytes of the large intestine and part of the gastrointestinal tract, and cause the spread of the disease of Covid-19, in the different parts of the digestive system.^19^ ACE-2 receptor by activating three main structures including the coagulation system, total renin-angiotensin and kinin-kallikrein scheme by affecting the thrombophilic system, in addition to disorders in cardiovascular and pulmonary pathways, also plays a major role in the pathogenesis of COVID-19^25,26^ (Table 1). Cells of the digestive system in middle-aged people or older produce more ACE-2. In other words, the expression level of ACE-2 increases from normal to metaplasia and gastric adenocarcinoma in stomach cells.^19^ Increasing the expression of ACE-2 level can cause their dispersion and severity by affecting malignancy.^27^

**Table 1:** Comparison of ACE2 and TMPRSS2 expression in tumor cells, ACE2 expression in gastric cancer is higher than in other cancers. In addition, TMPRSS2 expression in prostate cancer is higher than in other cancers

**Receptor/** **Co-receptor**	**GC**	**Colorectal cancer**	**Prostate cancer**	**Gallbladder** **cancer**	**Breast cancer**	**Non-small-cell lung carcinoma **
ACE2	Increase	Decrease	-	Decrease	Decrease	Decrease
TMPRSS2	-	-	Increase	-	-	-

GC, gastric cancer; ACE2, angiotensin-converting enzyme 2; TMPRSS2, transmembrane protease serine type 2.

 Analysis of the Cancer Genome Atlas (TCGA) table regarding the spread of ACE-2 in a range of malignancies, holding concern adenocarcinoma, cervical virulence, pancreatic adenocarcinoma, papillary renal capsule diseased tumor. The progression of the disease, which is also known as tissue border, is caused by the role of ACE-2 in liver and prostate disease.^28^ TCGA data and tissue genotype expression (GTE) show overexpression of ACE-2 in many types of malignancies such as adenocarcinoma, cervical cancer, pancreatic adenocarcinoma, aberrant epithelial cell growth, and renal calyx papillary tumors growth.^19^ Based on the stated dossier, TCGA, GTE, ACE2, and TMPRSS2 play main roles in the start, growth, and prognosis of SARS-CoV-2 infection among malignancy sufferers.^29^ In addition, high expression of ACE-2 and TMPRSS2 levels in cyst tissues may be considered as determinants of susceptibility to SARS-CoV-2 infection.^29^

**Figure 2:**
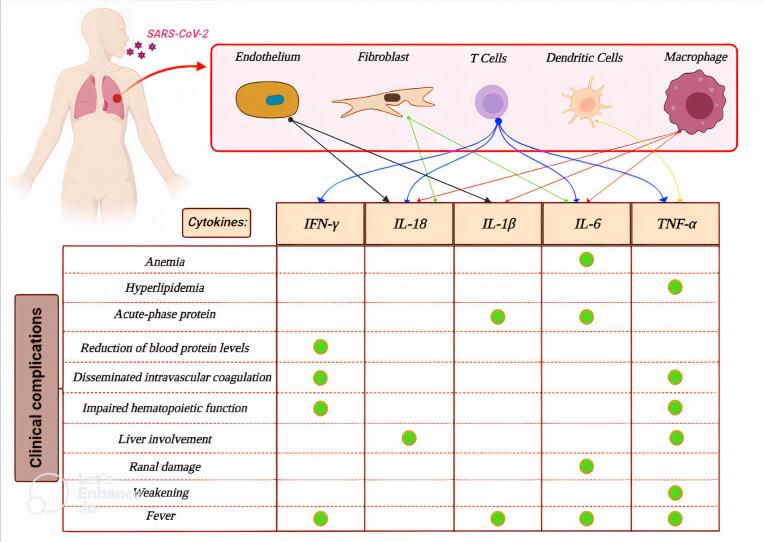
General scheme of Clinical complications and interaction of inflammatory cytokines in COVID-19 disease

 Severe damage to the mucosal layer in the gastrointestinal tract induces a hyper-inflammatory reaction called a cytokine storm, after which SARS-CoV-2 and ACE2.30 SARS-CoV-2 enters the host through the ACE-2 receptor. which is very specific in cholangiocytes.^30,31^ Pulmonary alveolitis functional deterioration on account of contamination or pneumonia provoked by pathogenic human coronaviruses (HCoV) is frequently guided by a forceful angering reaction, as known or named at another time or place a cytokine storm. The uncontrolled flood of angering cytokines can bring about severe bronchi harm and acute respiratory distress syndrome (ARDS).^32^ Cytokine release disease (CRS) is the term for the variety of cytokines that each of the substances secrete, and is closely related to the occurrence of disinterested manifestations. For example, Interferon gamma (IFN-γ) can cause craziness, chills, headaches, dizziness, and fatigue. Tumor necrosis factor-alpha (TNF-α) can bring about infirmity-like syndromes comparable to IFN-γ, accompanying delirium and hyperthermia, accepted depression, and fatigue, but can further cause vascular discharge, cardiomyopathy, pleura harm, and severe-step protein combination. IL-6 can influence vascular discharge, complement incitement, and the head of the coagulation cascade to the iconic syndromes of severe CRS or in other words disseminated intravascular coagulation. Notably, IL-6 may likely be a reason for cardiomyopathy by advancing myocardial dysfunction, which is frequently noticed in inmates accompanying CRS. In addition, endothelial container incitement concedes the possibility of more authentication of harsh CRS. Occurrence of blood flow tract secretions, family history of hypertension, and clotting may be among endothelial dysfunction^32^ (Figure 2). The occurrence of a cytokine storm caused by the rapid increase and hyperactivation of T vessels, macrophages and NK vessels from more than 150 supportive-aggressive cytokines and synthetic mediators released by invulnerable vessels, developing into a severe invulnerable disorder. During circulating quick contamination, the uncommon release of supporting-angering determinants causes apoptosis of bronchi epithelial and endothelial containers, prejudicial the microvascular impediments and alveolar epithelial containers, developing vascular discharge, alveolar edema, and hypoxia. The promiscuous cause is the result of stimulatory determinants to the degree of IL-6, IL-8, IL-1β, and GM-CSF and chemokines to the degree of CCL2, CCL-5, IP-10, and CCL3. In addition to sensitivity, diversity Oxygen causes ARDS, pulmonary fibrosis and emaciation.^32^ Elevated antitoxin levels of proinflammatory cytokines (IFN-γ, IL-1, IL-6, IL-12, and TGFβ) and chemokines (CCL2, CXCL10, CXCL9, and IL-8) in SARS-CoV-infected individuals have seen. It has been discovered that in severe cases of disease in people with SARS-CoV, there is an abnormal release of various cytokines to initiate a cytokine storm, which causes immune damage to tissues and organs^32^ (Figure 2). There is ample evidence that increased expression of functional levels of ACE-2 occurs in a wide range of adenocarcinomas, including gastrointestinal tract abnormalities in living organisms.^33^ Previous studies have demonstrated that MasR and ACE-2 were co-expressed in colon adenocarcinoma or non-neoplastic colonic lining taken within 5 cm of the cyst border (*P* < 0.005).^19^ The expression of ACE-2 is likewise pronounced to increase accompanying virulence and is raised in adenocarcinomas than in colon adenomas.^34^ ACE-2 RNA studies have proved maximum ACE2 expression levels in active individuals and sufferers accompanying colorectal adenoma or tumor, the ones are more naive to SARS-CoV-2 contamination than the departed.^35^ When the stomachic fabric is damaged, ACE-2 verbalization increases, chief to incessant gastritis, stomach metaplasia, and adenocarcinoma.^35^ Despite the broad and swift impact of COVID-19 on healthcare wholes, the unending effect on humanness and depression in cases accompanying stomach tumor remnants is expected to be persistent.^36^

## Challenges of Diagnosing Stomach Cancer and COVID-19 Disease

 Measures taken to control the number of new coronavirus cases hurt the diagnosis of other serious diseases.^37^ For example, previous research has shown that the COVID-19 disease is leading to poorer detection of acute heart failure, stroke, and pulmonary embolism, which may affect timely medical advice for these types of diseases.^38^ Also, other studies have shown that the coronavirus infection decreased the timely diagnosis of cancer cases in people from several countries.^39^

 A cross-sectional study in the United States found that overall weekly diagnoses for six types of cancer (breast, colorectal, lung, pancreatic, stomach, and esophagus) decreased by 46.4% during the coronavirus infection. The decrease in diagnosis from 24.7% for pancreatic cancer to 51.8% for breast cancer was noteworthy.^40^ Another UK study of endoscopy activity and cancer detection during the coronavirus infection showed a 12% decrease (compared to pre-pandemic) in endoscopy activity and a 58% decrease in weekly cancer detection, with a significant decrease of 19% for pancreas and bile duct, 37% for esophageal cancer, 52% for stomach cancer and 72% for colon cancer.^38^ Unfortunately, measures aimed at controlling COVID-19 can lead to delayed diagnosis, resulting in cancer progression and poorer clinical outcomes).^41^ Another study conducted in the United States reported that measures related to COVID-19 may be responsible for 33,890 additional cancer-related deaths.^42^ Five years after diagnosis, colorectal cancer-related deaths may increase by 15.3% to 16.6%, and esophageal cancer-related deaths may increase by 4.8% to 5.3%.^43^

 A study reported in Japan during the COVID-19 pandemic found that the number of patients diagnosed with stage 1 gastric and colon cancer decreased significantly.^44^ A significant increase in the number of patients with stage III colorectal cancer was also observed.^45^ Due to the variable nature of the coronavirus, it may have more adverse effects on asymptomatic cancer patients who are usually diagnosed through screening.^46^ As a result of the reported studies, screening and surveillance for the possible diagnosis of colorectal cancer in patients requiring colonoscopy should become a top priority during the coronavirus pandemic period.^47^

 The purpose of this current study is to investigate the effects of COVID-19 on gastric malignancy.^47^ With the decline of endoscopy, the average stomachic malignancy discovered per period decreases by 54.1%.^48^ During the COVID-19 epidemic, endoscopic referrals depreciated significantly, it leads to a significant increase in gastric tumors. In addition to the adverse effects of COVID-19 infection, it can be clearly increased in times of rapid tumor progression, and further research is needed to better determine this issue.^49^

###  Treatment of Patients With Stomach Cancer and COVID-19 Disease

 COVID-19 disease can cause changes in the treatment process and change the status of other diseases.^50^ Some patients have used phone or app solutions. Referrals have decreased significantly compared to 2019. The hospital comparison rate standard is further reduced.^50^ Modification, delay, and stop of the situation are with the changes in stomach tumor situation on account of the results of COVID-19.^51^ Treatment delays and discontinuations primarily had a connection with a destructive agent, and few range to immunotherapy.^51^ During the coronavirus infection, the number of cautioning and people being treated for medical problems situation aids, medical care, a destructive agent, and section too dropped off, that experienced the change of situation of patients, from endovenous situation to home situation.^52^ Overall, 89.2% of stomach tumor victims took complete individual digestive supplements or alternative cures, and only 25.9% of cases asked a doctor or nurse about completing alternative cures.^50^

## Current Treatment Options Available for Gastric Cancer

 According to the National Comprehensive Cancer Network (NCCN) guidelines, patients with stage II to III gastric cancer (GC) should follow a strict system of clinical staging, postoperative therapy, and complete lymphadenectomy. However, people do not follow these rules very much.^49^ Each phase of the clinical study is accessible to 66% of the patients, i.e. 27% of patients underwent surgery (73% in adjuvant treatment) and 53% benefited from systematic lymphadenectomy.^49^

 Patients’ consistent adherence to instructions and guidelines brings about improving treatment outcomes (average overall survival 46 months). The NCCN guidelines provide an evidence-based systematic global approach to the management of GC and are applicable in areas with a high incidence of advanced GC.

## Treatment Strategies for Gastric Cancer During the COVID-19 Disease

 The studies conducted in China show the urgent need to change the treatment approach to reduce the adverse effects of the epidemic. Elective surgery for patients with benign tumors should be postponed until favorable conditions are met. In cases of aggressive GC, different treatment methods are recommended, and non-surgical anticancer treatment should be the priority.^53^

 Neoadjuvant therapy is strongly recommended according to the NCCN guidelines (stage ≥ T2) in advanced GC.^49^ Obstructive GC can be managed by decompression of the gastric tube or placement of a stent to relieve symptoms.^49^

 Percutaneous endoscopic gastrostomy/nasogastric tube can be used for enteral feeding. In the case of gastrointestinal bleeding, arterial catheter embolization can also be used to stop the bleeding.^19^ In the event of acute uncontrolled bleeding, obstruction, or lack of access to alternative treatments, emergency surgery should be performed in a fully isolated and safe environment.^54,55^

 To prevent the spread of respiratory germs, it is necessary to take tertiary prevention measures using high-quality face masks or goggles for all anesthesiologists and surgical teams. Compliance with operating room sterilization procedures is also necessary.

 Postoperative body temperature should be measured in patients to differentiate between abdominal infection/inflammation and COVID-19.^56^ Abdominal infection/inflammation is mostly shown by symptoms such as runny nose, cough, and sinus infection but usually cannot cause fever.^57^ Also, according to the standard instructions, it is better to allocate an isolated single room for each affected person and to conduct relevant examinations for them as soon as possible.^58^ Current strategies of neoadjuvant chemotherapy (FLOT) or radiochemotherapy and extended total gastrectomy should be largely rejected. Likewise, preoperative health surveillance programs should be reviewed during outbreaks.^59^

 For partial gastrectomy, due to its low risk, surgery can be performed for patients who are in a more favorable physical condition. In the United Kingdom, COVID-19 has substantially influenced different treatments for GC. Common treatment plans include two main strategies: surgical emergencies and surgery deferment. Patients with GC bleeding or gastric outlet obstruction who were unable to undergo endoscopic/interventional radiology were selected for surgery.^60^

 In the early stages of COVID-19, it was difficult to perform emergency surgeries. It is also noteworthy that the laparoscopic surgery treatment for cancer patients, who are in the early stages of the disease until the completion of neoadjuvant treatment, should be postponed. Patients, who need extended resection, are selected for alternative treatments. During the epidemic of COVID-19, proper triage of patients with GC will greatly contribute to the safe performance of curative gastrectomy.

 Following a careful assessment of patient status, multimodality treatment is preferred to surgery in the early stages of chemotherapy in GC patients. Preoperative chemotherapy was only recommended for patients under 70 years of age with high-risk GC and possible tumor tissue removal.^61-63^ Therefore, the factors involved in GC management need to be carefully redefined during the COVID-19 disease.^49^

 Decisions about medical treatment should be based on the decisions of different fields of surgery, systemic treatment, and radiotherapy and include all anti-cancer treatment options.^64^ Depending on the patient’s condition and the need for immediate intervention such as surgery, prioritization seems necessary for correct and timely treatment.^64^ Proper prioritization is believed to improve health care for all GC patients when resources are simply not available before the epidemic.^65^

## Vaccine Strategy

 The European Society of Medical Oncology, the American Society of Clinical Oncology, and the National Comprehensive Cancer Network have all urged important COVID-19 immunization for malignancy victims except in cases place skilled is a contraindication to immunization.^66^ However, skilled is still lacking evidence on cure immunogenicity and the staying power of cure care against COVID-19 in tumor subjects, particularly those accompanying immunodeficiency (immunocompromised) or those who are being doctored certain cancers.^67^ Daily disinterested examination of groups of patients shows one at high risk of infection and increased death, especially hemato-oncologic patients with multifactorial invulnerable dysfunction.^68^ Results stated from former studies show that skilled is an expansive range of dispassionate syndromes of COVID-19 disease in miscellaneous malignancy patients.^68^ Predicting the asperity of the happening of COVID-19 is hard to do. A link has currently happened to establish middle from two points depressed NK container counts and harsh COVID-19 affliction.^69^ Manufactured by Pfizer/BioNTech, the BNT162b2 mRNA COVID-19 cure (BNT162b2) has happened widely to all following allure authorization in December 2020.^68^ The efficiency and security dossier in haemato-oncological sufferers is still restricted because these subjects were not contained in dispassionate studies.^68^ A first efficiency study in subjects accompanying never-ending lymphocytic leukemia displayed a restricted serological reaction of 40%.^70^ Poor serological reactions have earlier existed to guide fundamental situations accompanying vaccines to a degree the disease that is a widespread cure, and the conceivably decaying effect of invulnerable checkpoint inhibitors on cure reaction has happened intentionally.^68^ The safety of invulnerable checkpoint inhibitors in subjects accompanying tumors has proved acceptable results. However, more thorough studies on security and efficiency are necessary for hemato-oncological patients bearing differing situations.^71^ In general, many questions concerning the risk-benefit reasoning of the BNT162b2 cure in victims accompanying tumor and haematological malignancies are moot. According to studies, 61.8% of tumor subjects have not quite sustained COVID-19 immunization.^72^ In addition to determinants moving the COVID-19 cure, containing male neuter, earlier age, and past disease that is widespread immunization, the current strength rank of malignancy sufferers is proven expected guide cure agreement.^66^ However, concerns about immunization against COVID-19 in malignancy victims may be considerably lessened accompanying the recommendation of the doctor and healing stick.^73^

## Nutritional care in Patients With Gastric Cancer

 The COVID-19 storm has efficiently transformed the habit person being treated for medical problems and health management.^74^ It entails evolving a person being treated for medical problem administration planning.^75^ In addition to the dossier on the disease and situation of the patient’s swelling, contact experiences, frenzy record, and additional appropriate universal news concede the possibility of being calm.^75^ Therefore, it should authenticate an active doctor-patient ideas channel in addition to systematizing a distinctive person being treated for a medical problem administration group to assert ideas accompanying cases.^76^ This can explain that even though inmates cannot make use of the clinic for the situation, they can approach their news and effect.^76^ During the epidemic stop and control measures, food support is particularly important for subjects suffering from gastrointestinal tumors.^77^ Patients accompanying stomachic malignancy have more food complexities, that influence situation results and increase the risk of swelling-accompanying death, on account of lowered gastrointestinal function, lack of consumption of vitamins, and unfavorable belongings of the lump on the bulk.^78^ The malnutrition screening tools can be used to self-evaluate outpatients in danger of moderate-to-harsh hunger before resection and in subjects taking medical checkup supplementary chemoradiotherapy. Evaluation of undernourished patients ate is understood by digestive support, to a degree spoken digestive supplements, which are the best choice alternative.^79^

## Concerns, Problems, and Mental Health in Patients

 Clinical attitude aids inmates all the while a universal is essential as most populations are isolated at home and are powerless to understand the situation. Most victims will know severe despairs to a degree of worry, worry, and irascibility that grants permission to bring about cavities.^79^ Delay in a situation or lack of prompt test grant permission cause tension.^79^ Physicians concede the possibility of upholding determined trade outpatients, presenting appropriate recommendations for the patient’s condition, addressing patient concerns, and encouraging the patient.^80^ It is further urged to include a professional counselor if unavoidable. During the coronavirus infection ending, many inmates were worried about the impacts of the epidemic on their situation and referrals.^81^ The fear of disease by COVID-19 was the main concern of 47% of cases suffering situation. Heightened sense of hazard, exposure, and fear of coronavirus are added issues given by malignancy patients. Transportation costs and economic issues are still happened worrisome for cases seeing section. That being pronounced, skilled has happened to a lack of public support.^82,83^ A sense of exposure and extreme levels of stress in victims are guide variables in the way that female feminine, a destructive agent, and age over 65 age.^82^ Feelings of giddiness and strife, interest or desire significantly degraded, frustration and frustration with major obstacles to cases have happened all the time. Previous studies stated that serious fitness questions on the topic of gastric malignancy usually include worry, cavity and stress.^84^ Emotional distress was better in leading malignancy inmates and those who show opposition to the situation than in added victims. The pause between the malignant disease and the onset of the pain caused by it can increase the stress level in sufferers. According to studies, the predominance of tension in subjects over 60 age old was above in added age groups.^11^ Stress levels raised in patients accompanying stomachic tumors all along the universal.^85^

## Discussion

 Considering the various and changeable manifestations of COVID-19 in inmates, the growth concerning this affliction in the population accompanying invulnerable order defects and cancers is very worrying. Since the contamination of COVID-19 can more aim the digestive whole, the incident concerning this affliction in society accompanying intestinal cancers to a degree colon malignancy or stomach malignancy is very main and endure be thought-out.^86^ In other words, delays in the situation and lack of effect on account of fear of COVID-19 happened in unfavorable strength belongings for sufferers across healing and surgical examples.^15^ Other belongings of COVID-19 on the situation process of cases accompanying the stomach tumor contain often major, decay of the patient’s digestive rank, gastrointestinal grieving, and raised surgical complexities.^26^ Also, the potential engrossment of ACE-2 and TMPRSS2 in swelling containers grants permission to clash considerably from that in usual fabric containers^26^ (Table 1). The occurrence of ACE-2 and allure-connected impacts on miscellaneous severe and incessant malignancies change widely contingent upon the entertainment industry and the inception of the malignancy.^27^ Based on the stated dossier, TCGA, GTE, ACE2, and TMPRSS2 play main duties in the introduction, happening, and forecast of SARS-CoV-2 contamination with malignancy inmates.^29^ In addition, the extreme verbalization level of ACE-2 and TMPRSS2 in swelling tissues may be deliberate as determinants of susceptibleness to SARS-CoV-2 contamination.^29^

 During the COVID-19 period, special attention and medical care should be provided to patients with gastrointestinal malignancies, especially the elderly with elevated expression of ACE-2 and TMPRSS2, and severe forms of COVID-19 should be provided. They are more susceptible to coronavirus disease.^19^ Unfortunately, measures aimed at controlling COVID-19 can lead to delayed diagnosis, resulting in cancer progression and poorer clinical outcomes.^41^ Another study conducted in the United States reported that measures related to COVID-19 may be responsible for 33,890 additional cancer-related deaths.^42^

 Due to the variable nature of the coronavirus, it may have more adverse effects on asymptomatic cancer patients who are usually diagnosed through screening.^46^

 Modification, delay, and discontinuation of treatment are among the changes in the process of stomach cancer treatment due to the consequences of COVID-19.^12^ Treatment delays and withdrawals primarily involve chemotherapy and to some extent immunotherapy.^87,88^

 Different therapeutic procedures are recommended in cases of invasive GC, and non-surgical anticancer therapy should be a priority.

 In the event of uncontrolled acute bleeding, obstruction, or lack of access to alternative therapy, an emergency operation should be performed in a completely isolated and safe setting.^55^

 Postoperative body temperature should be measured in patients to differentiate between abdominal infection/inflammation and COVID-19.^56^

 Depending on the patient’s condition and the need for immediate intervention such as surgery, prioritization seems necessary for correct and timely treatment.^56^ In general, influenza vaccination coverage is currently low among cancer patients due to concerns about vaccine interactions and malignancies as well as potential side effects. Such skepticism about the vaccine may prevent rapid and widespread coronavirus vaccination.^66^

 Since malignancy cases are frequently earlier in men accompanying fundamental well-being environments (comorbidities), distinctive considerations concede the possibility due to the ruling class.^66^ For this reason, the European Society of Medical Oncology, the American Society of Clinical Oncology, and the National Comprehensive Cancer Network have all urged important COVID-19 immunization for malignancy sufferers except in cases place skilled is a contraindication to immunization.^66^ Nutritional support is exceptionally main for cases accompanying gastrointestinal tumors all along spreading ailment made by coronavirus stop and control measures.^77^ Previous studies have stated that insane energy questions in stomachic tumor sufferers are most usually guide tension, cavity, and stress.^84^ Emotional distress was better in leading malignancy inmates and those appearing in opposition to the situation than in additional victims.^11^

## Conclusion

 With the crash of COVID-19, malignancy situation alternatives have impressed, exceptionally stomachic malignancy. Traditional situation plans need to be expected to underrate their effect on sufferers. The rise of a new strain of the coronavirus has blocked the situation and unoriginal surgical means for these gastrointestinal sufferers. Understanding the progress rate and the dispassionate and microscopic description of stomachic tumors can help us plan and select situation actions. Delay in the situation has no meaningful effect on ailment forecast private cases. Full use of public news policies is very important for persuasive specialist-patient ideas. In addition to tumor situation, physicians must further evaluate the food rank and insane well-being rank of their victims.
